# Autoimmune response following influenza H1N1 vaccination in patients with juvenile idiopathic arthritis

**DOI:** 10.1186/1546-0096-9-S1-P133

**Published:** 2011-09-14

**Authors:** Nadia E Aikawa, Claudia Goldenstein-Schainberg, M Vendramini, Lucia MA Campos, Carla G Saad, Julio B Moraes, Alberto Duarte, Alex R Precioso, Maria CST Timenetsky, E Bonfá, Clovis A Silva

**Affiliations:** 1 Rheumatology, Universidade de Sao Paulo, São Paulo, SP, Brazil; 2 Adolpho Lutz Institute, Universidade de Sao Paulo, São Paulo, SP, Brazil; 3 Butantan Institute, São Paulo, SP, Brazil

## Background

To assess autoimmune response and H1N1 serology following influenza H1N1 vaccination in juvenile idiopathic arthritis (JIA).

## Methods

58 JIA patients were vaccinated with cepa A/California/7/2009 (NYMC X-179A) anti-H1N1. All participants received 1 dose of immunization, those <9yrs of age received a second dose 3weeks apart. All sera were evaluated before and 3weeks after complete vaccination. Serology against H1N1 virus was performed by hemagglutination inhibition antibody assay, antinuclear antibodies (ANA) by IIF, IgM and IgG anticardiolipin (aCL) by ELISA, and rheumatoid factor (RF) by latex fixation test.

## Results

Mean age of patients =23.9±9.5yrs, 38F:20M, mean disease duration=14.7±10.1yrs. Six(10%) had systemic, 33 (57%) poliarticular, 10(17%) oligoarticular and 9 (16%) other JIA subtype. Sixteen were off drugs and 42 (72%) under different drug treatments: 32(55%) using one DMARD/IS, 10(17%) on 2 DMARDs/IS, 19(33%) antimalarials, 29(50%) MTX, 8(14%) sulfasalazine, 6(10%) anti-TNFs, 4(7%) abatacept; no patient was using prednisone >0.5mg/kg/d. Seroprotection rates against H1N1 influenza increased from 23 to 83% and seroconversion rates were achieved in 78% JIA. Before vaccination 31(53.4%) of JIA patients were positive for ANA, 6(10.3%) for RF, and 4(7%) for both IgM and IgG aCL. After complete H1N1 vaccination, positivity for ANA remained the same whereas one patient became negative for IgG aCL, and another for RF, IgM and IgG aCL. Only 1/58 (1.7%) patient turned positive for low titer IgG aCL.

## Conclusions

Except for 1 patient that became positive for low titer IgG aCL, vaccination of JIA patients against pandemic influenza A (H1N1) generated successful protective antibody production with short term safety profile.

